# Person‐Centredness and Paternalism: The Dance With Power

**DOI:** 10.1002/msc.70032

**Published:** 2025-01-06

**Authors:** Clement Noel, Clair Hebron

**Affiliations:** ^1^ School of Sport and Health Sciences University of Brighton Brighton UK

**Keywords:** conceptualisation, French, person‐centredness, physiotherapists

## Abstract

**Objective:**

The World Health Organization advocates for person‐centredness (PC) as essential for quality care, yet its definitions and interpretations vary widely among professionals. Most qualitative research on PC focuses on physiotherapists in countries such as the UK, Australia, and the US, where PC is explicitly emphasised. In contrast, the term PC is absent in French educational standards, and its promotion is lacking in the French healthcare system. This study explores French physiotherapists' conceptualisation of PC.

**Methods:**

This phenomenographic study followed an interpretivist paradigm. Seven physiotherapists practicing in France were purposively selected. Data were collected through remote semi‐structured interviews, transcribed verbatim, and translated into English. The analysis followed the steps outlined by Larsson and Holmström (2007).

**Findings:**

Three categories were generated to illustrate the conceptualisation of PC by participants: *Creating a relationship, Adapting the rehabilitation*, and *Giving the choice*. Despite recognising the importance of these aspects, they also highlighted challenges related to paternalism and power dynamics, which often limited trusting relationships, effective adaptations and true shared decision‐making.

**Conclusion:**

While these physiotherapists expressed a commitment to person‐centred practices, they encountered challenges in relinquishing power, highlighting the ongoing journey towards becoming person centred.

## 1 Introduction

It is internationally recognised that person‐centredness (PC) is fundamental for quality healthcare. The World Health Organization (WHO) defines quality care as ‘*safe, effective, people‐centred, equitable, efficient, timely, and integrated*’ (WHO 2024). Institutions such as the World Physiotherapy 2011; the Health and Care Professions Council (HCPC) and the Chartered Society of Physiotherapy (CSP) in the UK; and the American Physical Therapy Association (APTA) in the US advocate for PC in their standards and educational frameworks (APTA 2018; CSP 2019; HCPC 2021). PC has also gained prominence in physiotherapy research, as reflected in an increasing number of publications (Jesus et al. 2021). It is a complex, multifactorial concept (Jesus et al. 2022), with research revealing diverse trends in its perception (Hansen, Præstegaard, and Lehn‐Christiansen 2022).

Scientific literature identifies three PC discourses. The first, ‘*caring for persons*’ emphasises gathering essential information and fostering adherence to advice. The second, ‘*empowering persons*’ focuses on shared decision‐making. The third, ‘*being responsive’* highlights tailoring health information to meet individuals' needs (Pluut 2016). These discourses span diverse fields, including healthcare ethics, sociology, education, and nursing, representing a range of disciplinary perspectives.

Despite extensive research, studies on physiotherapists' conceptualisation of PC remain scarce. PC is embedded in a culture shaped by political, cultural, economic, and historical factors, varying across countries (Hutting et al. 2022a; McCormack et al. 2015). Research has predominantly focused on nations such as the UK, New Zealand, Australia, and the US, with no studies addressing France (Hutting et al. 2022a 2022b; Mudge, Stretton, and Kayes 2014; Schmitt, Akroyd, and Burke 2012; Pluut 2016; Ward et al. 2018).

In France, PC is not explicitly advocated in government policies or health institutions. However, the 2007 Haute Autorité de Santé (HAS) document ‘*Together, Defining New Horizons for Quality in Healthcare*’ touches on elements of PC, such as involving patients in decisions and acknowledging illness experiences, though the term ‘PC’ remains rare in guidelines (HAS 2007). More recently, French reports have discussed PC in complementary medicine or public health, illustrating inherent paternalism in the medical system while suggesting efforts to enhance person satisfaction and care efficiency (Engberink et al. 2018; Sebai and Yatim 2018).

The French physiotherapy curriculum requires competencies in elements of PC, such as empathy, communication, therapeutic alliance, and autonomy promotion (Legifrance 2015). However, PC and shared decision‐making are absent in its detailed content, potentially limiting its emphasis. No studies have examined how French physiotherapists conceptualise PC. Given the unique context of French healthcare, this research aims to address this gap by exploring French physiotherapists' conceptualisation of PC.

## 2 Methods

### 2.1 Methodology

This study adopts an interpretivist perspective, which acknowledges that reality is subjective and socially constructed, with each person experiencing reality through the lenses of their own perspective. Consequently, individuals may have different understandings of the same concept, leading to variations in their actions (Nicholls, 2017a; Petty, Thomson, and Stew 2012). The study employs a qualitative phenomenographic approach, which takes a second‐order perspective exploring the world as it is understood, rather than how it is (Barnard, McCosker, and Gerber 1999). Phenomenography aims to capture a range of perceptions of a phenomenon and explore variations in meaning (Åkerlind 2005; Akerlind et al. 2005; Giorgi 1999; Marton and Svensson 1979; Yates, Partridge, and Bruce 2012).

### 2.2 Methods

Participants were purposefully recruited via an advertisement posted on *X* (Twitter) and through emailing the members of the French physiotherapy organisations SFP and OMT‐France. Seven physiotherapists who worked and were trained in France were recruited for the study. This is considered sufficient in phenomenography to provide a variety of perceptions while keeping the amount of data manageable (Trem 2017; Yates, Partridge, and Bruce 2012). To provide context participants were asked for background information (Table 1). They were then selected to ensure maximum variation in their training, workplace, post‐graduation training, experience, gender, ethnicity, and age. The rationale for this purposive selection was to gather a wide range of perceptions, providing depth and richness in experiences (Åkerlind 2005), as these criteria may lead to greater variety in worldviews (Morse 2009).

**TABLE 1:** Demographic information of participants.

Participants	Age	Sex	Graduation	Practice
Annie	30	F	2015	Private neuro MSK geriatric
Francois	26	M	2019	Private neuro MSK cardio + education
Michel	27	M	2017	Private MSK geriatric
Claudine	25	F	2021	Private neuro MSK geriatric + education
Dagobert	35	M	2009	Private neuro MSK
Nicolas	39	M	2004	Private neuro MSK geriatric paediatric
Martin	24	M	2020	Private MSK + research + other

Data were gathered using semi‐structured interviews, allowing some fluidity according to each interaction with a participant. The average interview duration was 59 minutes. Interviews were conducted remotely by the researcher, adhering to Covid‐19 restrictions using Microsoft Teams and audio and video recordings.

The interview guide was initially developed by the first author based on Kvale and Brinkmann (2015) recommendations for qualitative research interviews. This draft was then reviewed and collaboratively refined with the second author to produce the final version. In‐line with our methodological approach we came from a position of wonder, in which we were ready to be surprised. Thus, we held back our pre‐understandings and a structured guide was avoided. Instead, participants were encouraged to share concrete examples of their experiences with the phenomenon of interest, with prompt questions used to elicit richer, more detailed narratives. For instance, participants were asked, “Could you think back to a recent clinical encounter which you felt was person centred … take your time to take yourself back to that time…can you describe in as much detail as possible what made the encounter person centred … what did you do and how did you do it?”. Various alternative ways of asking about the phenomenon of interest we used for example; “thinking back to a particular encounter, maybe a quite different encounter… or an encountered which wasn’t so person centred…can you describe that to me in as much detail as possible?” Prompts such as, “can you describe that to me in more detail” “Can you give me an example of that?” or “You mentioned *X*; can you tell me more about it? … what did you do at that time and how did you do it?” were used to encourage deeper reflections. The complete interview guide is provided in Figure S1.

## 3 Data Analysis

Following the steps of analysis described by Larsson and Holmström (2007), interviews were transcribed verbatim, anonymised using pseudonyms, and translated into English by the first author, a French musculoskeletal physiotherapist working in the UK. The author chose to denaturalise the quotes by removing pauses, false starts, filler words, silences, repetitive phrases, overlapping talk, or non‐response tokens, and correcting grammar or adding linking words and commas to ensure readability. The transcripts were read multiple times to familiarise the researcher with the data. Passages that addressed the research question were highlighted. These fragments of text were then broken into meaning units (sections of text containing one meaning). The researcher initially examined ‘what’ participants were talking about (the focus of their attention) and ‘how’ they described it. This led to a preliminary description of participants' conceptualisation of PC. These descriptions were grouped into categories based on their similarities and differences (Giorgi 1999). Predominant categories of description were illustrated by a title. Finally, non‐dominant ways of understanding PC were also examined to explore variation. The writing process was an inherent part of the analysis (Pierre and Elisabeth 2015). Indeed, while writing, the researcher identified categories with varying depths and their main characteristics. The second author, an experienced academic, qualitative researcher and physiotherapist, offered alternative interpretation and entered into dialogic discussion with the first author at each stage of the analysis.

## 4 Findings

Three categories of descriptions represented participants' conceptualisation of person‐centredness: *Creating a relationship, Adapting the rehabilitation*, and *Giving the choice*.

### 4.1 Creating a Relationship

Participants in this study described creating a supportive relationship as an important aspect of person‐centredness (PC). The characteristics of this relationship included friendliness, trust, support and communication. The attributes of this relationship varied among participants: some expressed that a more friendly and warm relationship helped develop trust and gain insights into the person's world, while others spoke of maintaining a professional distance.

I was a student; we had a different relationship together than what we could expect between a physiotherapist and a patient. We talked straight to the point, and it was a warmer relationship, a kind of friendship. This is why she talked to me about her experience of the handicap. (…) This, other therapists could not access. (Francois)

We have created bonds, purely professional ones, but she trusted me about a lot of things. I knew a lot about her, I knew her ways. (Claudine)

Participants conveyed the central importance of communication skills such as listening and expressing empathy.

I tried to listen to her complaint, to say that I understood that it was difficult. I supported her and listened to her complaint. (Dagobert)

I tried to dig into the psychosocial aspect of the person by open questions, I used active listening, communication tools, motivational interviewing, I included reflections, open questions, reformulations, or summaries of what they said. (Claudine)

Communication was not only verbal but also non‐verbal. For example, Claudine described how she used space to create a sense of safety and facilitate communication.

We went to my office, I made her sit, and I sat in front of her, at the same height. I was not behind my desk, there was nothing between us and I was just sitting on another support. I thought it was important. (Claudine)

In crafting the relationship, several participants expressed struggles with managing their own emotions and judgements. Their descriptions revealed a sense of hierarchy and authority.

He did not explain the situation, he refused to tell me what was going on with him (…) Not giving up was an effort, it was an immense effort. Regarding why it didn’t go well: I think if the person was kinder, less aggressive, I would have enjoyed helping him more and I may have made more efforts, done more research, I don’t know … I would’ve found more ways to help him … I think I sometimes was less involved in comparison to someone who would have not showed up in such a bad way during the first session (Annie)

When I saw him, and he showed me the scratch on his knee, I said ‘ohhhh it's like for children, a little kiss on the knee and it's all good.’ I said it nicely, with a smile, to kind of teach him, because from the beginning, I kept repeating to him that his knee is not fragile, that it will not break. It was a way through a joke with a smile, to make him realize the gap between what happened, and the pain that he experienced. (Nicolas)

Participants described creating a positive therapeutic alliance as an important element of PC, which provides a basis for collaboration and *Adapting the rehabilitation*.

### 4.2 Adapting the Rehabilitation

Another category that emerged from the participants' interviews was the need to adapt the treatment, rehabilitation and (albeit less frequently) the assessment process to the individual. A key attribute of *Adapting the rehabilitation* involved tailoring to the person's needs, preferences and lifestyle, including their activities, job, and other personal factors.

I adapted to what she told me, and we really found solutions that were adapted to her problems and her daily life. I relied on the activities she was doing at work, at home, what she could not do anymore, what she wanted to do again. (Michel).

I was able to adapt to what seemed to me the best for her, find which test was relevant, then adapt and customise the exercises to her life. (Martin)

Some transcripts illustrated the importance of *Adapting the rehabilitation* to help the person navigate social situations while living with a disability. For example, they highlighted the significance of respecting a person's wish to change the goal of rehabilitation due to the social normative view of disability, which can diminish the perceived legitimacy of their handicap.

As she knew that her hand and foot would not recover fully, she wanted her handicap to remain visible to avoid having to explain and argue about her invisible handicap in public transport. (Francois)

Despite the emphasis on A*dapting the rehabilitation* to the individual, many examples of "usual care" were provided, where the physiotherapist applied standard practices regardless of the specific person they were treating.

I did a Mackenzie assessment to begin with, because I'm certified in McKenzie, and I always start with it for people who come for back problems whether it looks mechanical or not. (Michel)

One participant described the adaptation of their exercise prescription as person‐centred by mimicking the tasks of the person’s occupation and therefore making it “*meaningful*”. However, the participant's wording suggests it may be meaningful to the physiotherapist rather than the person.

It was person‐centred because I had given meaning to the exercise. As she was a housekeeper, and she told me that lifting a stool was difficult for her, I gave her dumbbells to lift. I gave her something which, for me, was person‐centred because it was meaningful. (Dagobert)

Some participants felt that person‐centredness was reflected in their ability to adapt the rehabilitation to the person considering the biopsychosocial model rather than focussing on the disease. However, some participants expressed a reductionist understanding of the biopsychosocial (BPS) model, limiting the adaptations to their perceptions.

So, for me, my approach was person‐centred, in the sense that I tried to dig into the psychosocial aspect of the person. (Claudine)

In the past, I accepted the social role a little bit. Now, I'm not willing to accept this social role. I am ok to accept a psychologist role, but not a social role. (Michel)

One participant understood person‐centredness (PC) as a continuous adaptation of rehabilitation to evolving needs. Claudine illuminated this in her description of changing her plan to safeguard a person suffering from depression with suicidal thoughts. Initially, the participant intended to discharge the person from their care, yet they continued treatment for a few more sessions, focussing on monitoring the person's mental health.

The patient told me she was thinking about death. It then became a safeguarding issue, as in a week it changed completely. She really was not like that initially. (Claudine)

### 4.3 Giving the Choice

While the category of *Giving the choice* was present in most participants' accounts, its meaning varied. Some participants acknowledged struggles with navigating control and provided examples where they maintained control over the rehabilitation process by using directive vocabulary and exhibiting a more biomedical posture. Similarly, another participant explained how they tried to manipulate the person to achieve their desired outcome.

From the very beginning I told her that we would do active rehabilitation for her shoulder, with strengthening. I identified the external rotators and the upper trapezius with a deficit. So, I explained that I was going to work her shoulder that way. (Nicolas)

Trying to listen as much as possible and kind of manipulate him to make him express things that are stuck, to guide him towards expressing, at least in some degree, this unpleasant situation (Annie).

One participant expressly explained the difficulty of sharing the responsibility and decision‐making with the person.

The problem with person‐centredness is that sometimes it is very difficult to know how much space to give the person. In theory, the person should be responsible for their care, because it is their problem, so their vote should be the one influencing the decision. However, in the Evidence Based Practice model EBP, the person only accounts for a third of the model. Sometimes, when a patient is 100% in wrong beliefs with numerous biases, it is difficult to leave them at the centre and remain in a horizontal system (Francois).

The boundaries of person‐centredness were also experienced differently. For example, one participant felt that the partnership was not part of person‐centredness but was beyond. For them, person‐centredness is limited to focussing on the person.

With the “patient as a partner approach”, we are beyond patient centredness, beyond the fact that we focus on the person, but instead we work in partnership with them. Therefore, the person is not at the centre of the medical and health team but is part of the team. (Martin)

Others placed emphasis on respecting the person's choice and were aware of the power they held as physiotherapists and endeavoured to mitigate it, allowing the person to be in control or give direction.

She was happy not to be directed or ordered what to do during the rehabilitation process. (…) She did not like when physiotherapists or occupational therapists told her to do things. With me it was more a collaboration compared to what she was used to (…) She was deciding what to and not to work on. (Francois)

I believe that taking sides is to be somewhat directive and I really do not want to be directive. (…) I don’t want my words or my status to influence her decisions in a way or another, knowing that I am not her. I can’t be in her shoes; I can’t live what she is going through etc. So that’s why I don’t want to take the lead or the least I can. (Claudine)

To conclude the findings, it is crucial to highlight the interconnected nature of the three categories—*Creating a relationship, Adapting the rehabilitation, and Giving the choice*. These elements do not operate in isolation; instead, they are interwoven with the dynamics of power threading through each category. The therapeutic encounter requires constant navigation and negotiation of power, with physiotherapists balancing their expertise with the individual's preferences, autonomy, and evolving needs. This interplay resembles a dance, where the roles of guiding and following are shared, fluid, and dynamic, setting the stage for person‐centredness.

## 5 Discussion

This study aimed to explore how French physiotherapists conceptualise person‐centredness (PC) within their practice. Through the analysis, three categories of description were generated: *Creating a relationship, Adapting the rehabilitation*, and *Giving the choice*. In this discussion, we have used the metaphor of a dance with power, where clinicians balance the intention to support the person with the complexities of decision‐making and control. In some dances such as Swing or Lindy Hop, power circulates between the ‘lead’ and the ‘follow’. Unlike in other dances, the lead suggests moves, but the follow is free to decide what they want to do with the guidance provided. An effective lead guides the follow through a sequence of steps, actively listens, and fosters the follow's creative expression, cultivating a dynamic partnership grounded in mutual respect and adaptability (Trommer‐Beardslee 2022). A lead that rigidly controls every move risks stifling the artistry of the dance and disengaging the follow from the process. These delicate balances within a dance partnership reflect the collaborative relationship between physiotherapists and patients, echoing the findings of this study and highlighting broader tensions surrounding power dynamics in physiotherapy (Gibson et al. 2019).

### 5.1 Challenges of Paternalism: The Dance With Power

The category of description *Creating a relationship* highlighted participants' struggles in establishing and maintaining trusting therapeutic relationships. This echoes findings among Swedish physiotherapists in acute care, where trust was often unidirectional expected from patients but not always reciprocated by clinicians struggling to acknowledge the power dynamic of the relationship (Sjöberg and Forsner 2020). In our study, such asymmetry in relationships occasionally led to patients being blamed for breakdowns or led to a paternalistic approach when they questioned clinicians' authority or struggled with treatment adherence, as illustrated by the quotes from Annie when she blamed the person for not explaining why they were not keen on sharing information with her negatively impacting their relationship. This behaviour aligns with Talcott Parsons' ‘sick role’ concept in which individuals are expected to conform to certain behaviours (seek assistance and comply with advice provided) (Nicholls, 2021; Parsons 1952) potentially undermining PC (Jesus et al. 2022). The notion of ‘compliance’ or ‘adherence’ while closely tied to person‐centredness (PC), underscores an inherent paternalism that subtly positions the clinician as the ultimate authority and the patient as a passive recipient of care, creating tension with the collaborative ideals of PC (Ward et al. 2018).

This category, C*reating a relationship,* resonates with the therapeutic alliance a concept interrelated with PC. The literature highlights elements such as clinician presence, empathy, and effective communication strategies, including active listening as pivotal in creating good alliances (Hutting et al. 2022a; Jesus et al. 2022; Miciak et al. 2019; Søndenå, Dalusio‐King, and Hebron 2020). The power asymmetries evidenced in our findings are inherent in healthcare due to disparities between medical knowledge and patients' knowledge, which often perpetuate epistemic injustice (Carel and Kidd 2014). These dynamics are frequently underpinned by paternalistic attitudes, where clinicians position themselves as the ultimate authority on health, sidelining patients' lived experiences and agency (Murgic et al. 2015). Furthermore, societal expectations often privilege medical expertise over personal narratives, reinforcing hierarchical structures and creating barriers to mutual understanding (Illich 1976). Arguably dancing with power within a relationship is crucial for fostering and maintaining trust, as both parties share responsibility. This requires clinicians to move beyond paternalism and embrace relational approaches, where trust is co‐constructed, and responsibility is shared. This adaptive approach reflects Gibson et al.'s (2019) concept of ‘tinkering,’ emphasising the dynamic process of refining care within established protocols and power structures.

All participants in the current study acknowledged an imperative to be person centred in their practice and in this highlighted the importance of *Giving the choice*. This resonates with shared decision making (SDM) a concept interrelated with PC (Hutting et al. 2022b; Coulter and Collins 2011). SDM is a collaborative process where clinicians and individuals clarify treatment goals, exchange information about options and desired outcomes, ultimately agreeing on the most suitable course of action (Coulter and Collins 2011). Although this varied for all participants in the current study, there was a sense of navigating power when making decisions related to management and treatment. While some participants' accounts gave a sense of effective SDM, in some cases, participants described adopting a directive paternalistic stance where they made decisions based on their knowledge without consulting individuals' preferences. Some participants also described giving limited choices which were based on their own preferences, biases and a prevailing ‘I know best’ mentality with the aim of achieving the desired outcomes. This reflects a broader tension between the ideals of PC and the prevailing paternalistic practices within the profession (Sjöberg and Forsner 2020; Sullivan, Hebron, and Vuoskoski 2019). Such control can undermine the principles of PC, suggesting a lack of trust in individuals' ability to make competent decisions about their care, hence the suggestion of a tinkering approach with humility and flexibility (Gibson et al. (2019). Similarly, other participants described decisions that weren't shared, but in which patients were made to choose even when they explicitly indicated a lack of preference or a desire not to share in the decisions. This contrasts with literature advocating for flexibility in involvement, aligning with individuals' preferences and readiness to engage (Jesus et al. 2022). However, the literature suggests that according to specific contexts, physiotherapists may limit options under the guise of benevolence and that whether it would still be PC could be debatable (Gibson et al. 2019). Echoing Tuckett's (2006) definition of paternalism as a ‘*benevolent decision‐making in another's best interests’.* The additional layer of challenge and complexity lies in clinicians dancing with the circulation of power during the encounter to explore the individual's desire for involvement in starting to consider that better choices might be made if they can bring their specific context and circumstances into the equation.

These French physiotherapists described *Adapting the rehabilitation.* This included navigating cultural and professional boundaries and influencing their practice adaptations. However, they described these modifications as being constrained by their perceptions of standards of appropriate care. This illuminates the power of evidence‐based practice (EBP) and clinical guidelines, which, while crucial for standardising care and ensuring consistency in treatment, can inadvertently constrain health choices by prioritising scientific evidence over individual patient preferences, cultural contexts, and lived experiences. This reliance on guidelines can result in a one‐size‐fits‐all approach, diminishing the flexibility needed for person‐centredness and potentially reinforcing power imbalances in clinician‐patient relationships (Greenhalgh et al. 2014; Gabbay and May 2004). Although the guidelines focus on objective medical factors, participants in this study also considered the broader psychosocial context when *Adapting the rehabilitation.*

Participants descriptions included references to the BPS model, which underscored its perceived relevance to PC. However, their accounts revealed variations in its conceptualisation and operationalisation as illustrated by Michel's quote: ‘*I'm not willing to accept this social role. I am ok to accept a psychologist role, but not a social role*’. This echoes criticisms of the fragmented and somewhat ‘biomedicalised’ interpretations of the model, which lacks a focus on the social and sociological (Daluiso‐King and Hebron 2022; Mescouto et al. 2020; Cormack et al. 2023; Roberts 2023), often neglecting the social determinants of health, which risks oversimplifying the complex interplay of structural inequalities and societal influences (Wade and Halligan 2017). This aligns with critiques of PC, which, despite its merits, can marginalise structural awareness by focussing on individual agency while neglecting the broader neoliberal forces shaping healthcare priorities and resource allocation (Mol 2008; Wilkinson and Pickett 2009). What was missing from the data was attention to broader micro‐ and macro‐environmental factors, including the roles of significant others, team members, and the structural organisation of PC practices (Jesus et al. 2022) or culture, economics, environment, politics. One potential strategy for addressing this is to draw on Anne‐Marie Mol's work on ‘Multiplicity’ (Setchell, Nicholls, and Gibson 2018). Mol's conceptualisation advocates for recognising pluralism of ontologies—understanding entities are enacted and perceived in various dynamic, context‐dependent ways across diverse social and cultural landscapes. By adopting this perspective, clinicians may enhance their flexibility and overcome perceived barriers to adapting practices to individuals. Just as exceptional dancers acknowledge their partners' uniqueness and adapt to their styles, preferences, and experiences, so too can clinicians refine their approaches to optimise care.

This discussion has illustrated the dance of power inherent in person centred practice. Traditionally, physiotherapy has operated within a paternalistic biomedical model, reducing individuals to mere subjects of study, thus diminishing their uniqueness and framing experiences within standardised patterns of care and resisting person‐centredness (Nicholls, 2017b; Nicholls, & Gibson, 2010; D. A. Nicholls, and Gibson 2010; Kvåle and Bondevik 2008). Power is complex, with its circulation—or lack thereof—often perceived as oppression, as articulated in Levinas' terminology, insofar as unequal power dynamics can limit individuals' agency, reinforcing hierarchical structures that hinder person‐centredness (Maric and Nicholls 2020). Just as in dance, where both partners contribute to the flow, person‐centeredness required tinkering with power when *Creating relationships, Giving the choice and Adapting the rehabilitation*.

In navigating the complex dynamics of power within the therapeutic relationship, clinicians and patients engage in a dance with power, where the balance of authority, knowledge, and autonomy is continually negotiated. As Foucault (1980) asserts, power is not inherently oppressive but can be productive and transformative when wielded thoughtfully, emphasising that power's subtle and omnipresent nature can foster relationships built on collaboration, respect, and mutual agency, rather than mere domination. In this sense, power in the therapeutic context, when shared and acknowledged, becomes an opportunity for growth, rather than a force of control.

### 5.2 Limitations

This study has several limitations. First, the first author was a novice interviewer, which may have impacted the depth and nuance of the interview data (Kvale and Brinkmann 2015). Nonetheless, the interviews yielded diverse and valuable insights. Second, there was a selection bias, as all participants demonstrated familiarity and interest in person‐centredness (PC), potentially excluding perspectives from those less engaged with the concept. Seven participants were included—which is considered sufficient for phenomenographic research (Trem 2017; Yates, Partridge, and Bruce 2012). Phenomenography aims to capture a range of conceptualisations and does not seek saturation (Barnard, McCosker, and Gerber 1999; Braun and Clarke 2019; Saunders et al. 2018); thus, we do not claim data saturation; the findings reflect these participants' conceptualisations at this point in time and are not exhaustive. Lastly, qualitative research often faces scrutiny over researcher bias. Both researchers are MSK physiotherapists, with the second author being an experienced qualitative researcher. Phenomenography recognises the co‐construction of meaning between participants and researchers (Yates, Partridge, and Bruce 2012), and methodological transparency has been maintained to allow readers to assess the trustworthiness of the process (Kvale and Brinkmann 2015).

#### 5.2.1 Implications

The findings highlight the need to integrate awareness of power dynamics into physiotherapy education, practice, and policy. Reflective practices are crucial for clinicians to identify and address biases, fostering a shift beyond paternalistic approaches. Educational programs should emphasise relational and communication skills alongside flexibility in care delivery, encouraging clinicians to navigate the diverse interpretations of person‐centredness (PC).

Future research could examine PC in settings where it is institutionally prioritised or explore how changing societal and professional values shape its application. By adopting humility, benevolence, and adaptability, physiotherapists can foster PC.

#### 5.2.2 Conclusion

This study contributes to the understanding of person‐centredness (PC) within an underexplored cultural and professional context, highlighting how French physiotherapists conceptualise and navigate PC in their practice. The findings revealed three interconnected dimensions—C*reating a relationship, Adapting the rehabilitation, and Giving the choice*—that illustrate the nuanced nature of PC as experienced by participants. However, power dynamics, often framed through paternalistic attitudes, emerged as a persistent thread, shaping and complicating the enactment of PC principles.

The metaphor of a ‘dance with power’ encapsulates the ongoing negotiation required in therapeutic encounters. While participants demonstrated a commitment to supporting individuals' needs and preferences, the interplay of guidance, autonomy, and relational input revealed tensions. These dynamics often reflected a benevolent paternalism, where clinicians' intent to guide care sometimes limited individuals' agency and trust. Recognising this balance as an adaptive, dynamic process rooted in respect and collaboration provides an opportunity to refine approaches to PC, making them more inclusive and context‐sensitive.

## Author Contributions

The first author conducted all the interviews, while both authors collaboratively developed the research question, methodology, and methods. They jointly analysed the data and contributed to writing and refining the article.

## Ethics Statement

Ethical approval for the study was granted by the School of Sport & Health Science Research Ethics and Integrity Committee of the University of Brightonon 15 December 2021.

## Conflicts of Interest

The authors declare no conflicts of interest.

## Supporting information

Figure S1

## Data Availability

The data that support the findings of this study are available from the corresponding author upon reasonable request.
