# Supplementary material for: Person‐Centredness and Paternalism: The Dance With Power
Source: Musculoskeletal Care. 2025 Jan 6;23(1):e70032. doi: 10.1002/msc.70032 (PMC11704454; doi:10.1002/msc.70032)
Supplement: Supplementary file 1 — Figure S1 [file MSC-23-e70032-s001.docx]

## **Supplementary Figures**

**Figure 1.**  Interview Guide

| **Before the interview**  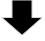 |
| --- |
| 1. *Do you have any questions regarding the consent form or the information sheet?* 2. *I would like to remind you that you are free to withdraw at any time. If you decide to do so during the interview, the already recorded information may still be used.* 3. *Although I am a physiotherapist today, I am here as a researcher, so please don’t assume I have any physiotherapy knowledge* 4. *The interview is not a normal conversation, it serves the purpose of exploring your experience of person-centredness. There are no correct or wrong answers, and I am not looking for anything in particular. It is just about exploring your experiences and perceptions* |
| **During the interview**  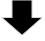 |
| **(Introductory questions, open ended)**  *Could you think back to a recent clinical encounter which you felt was person centred … take your time to take yourself back to that time…can you describe in as much detail as possible what made the encounter person centred … what did you do and how did you do it?*  *Thinking back to a particular encounter, maybe a quite different encounter…. or an encountered which wasn’t so person-centred …can you describe that to me in as much detail as possible?*  **(Prompts)**   - Reflect what was said meaningful / significant words (unusual terms, intonations) - “Hmm” - Nodding - Silence - Thank you for that - Take your time   **(Probing questions, seek clarification)**  *Could you tell me more about that?*  *Can you give me more details of what happened?*  *Could you give me examples of what you are saying?*  *Do you have further examples of this?*  *What happened?*  *What did you do and how did you do it?*  *What did the patient say / do?*  *What do you mean by ”…”*  **(Delayed questions)**  *Would you be happy to come back to … ?*  *You mentioned a lot of interesting things, I would like to come back to a few of them to explore them in more detail, is that ok for you?*    **(Additional topics & summary)**  *Is there anything else that you would like to add to explain your understanding of this concept?* |
| **End of the interview**  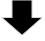 |
| *Thank you for your willingness to share your experiences. This has been very helpful.*  *I have no further questions. Is there anything else you would like to bring up, or ask about, before we finish the interview?* |
